# The duration of chronic low back pain is associated with acute postoperative pain intensity in lumbar fusion surgery: a prospective observational study

**DOI:** 10.1186/s12871-022-01674-w

**Published:** 2022-04-29

**Authors:** Mei-ping Qian, Mei-rong Dong, Juan Li, Fang Kang

**Affiliations:** Department of Anesthesiology, The First Affiliated Hospital of USTC, Division of Life Sciences and Medicine, University of Science and Technology of China, Hefei, 230036 Anhui China

**Keywords:** Duration of chronic low back pain, Acute postoperative pain, Lumbar fusion surgery

## Abstract

**Background:**

Pre-existing chronic pain has been associated with severe postoperative pain. In this study, we sought to prospectively analyse the association between the duration of chronic low back pain and the intensity of acute postoperative pain after lumbar fusion surgery.

**Methods:**

A total of 330 patients who underwent lumbar fusion surgery were divided into three groups (chronic low back pain less than 1 year, one to 5 years, and more than 5 years) based on the duration of chronic pain. On the first postoperative day, the maximum pain scores of each patient were recorded during the day and at night. Bivariate correlation and logistic regression were performed to identify relationships between acute postoperative pain and related variables (age, sex, smoking history, body mass index, operation history, duration of surgery, level of preoperative pain, aetiology of back pain, Self-rating Anxiety Scale, Self-rating Depression Scale, FRAIL scale, and duration of chronic low back pain). If the postoperative pain score was > 3 when the patient reported was at rest, the patients were treated with postoperative intravenous self-controlled analgesia or rescue analgesics if necessary.

**Results:**

There was an association between severe acute postoperative pain and the duration of chronic low back pain. In terms of VAS day, multivariable logistic regression showed the duration of chronic low back pain was not statistically significant (OR = 2.48, 95% CI: 0.900 to 6.828, *p* = 0.0789). The result is uncertain because the confidence interval included the null after controlling for SAS, SDS, BMI, and aetiology of back pain. In terms of VAS night, patients with a duration of chronic low back pain of more than 5 years were more likely having moderate to severe acute postoperative pain (VAS > 3) compared to patients with a duration of chronic low back pain less than 1 year (OR = 3.546, 95% CI: 1.405 to 8.95, *p* = 0.0074). Hospital stay, the pain score on the day of discharge and the pain score after 3 months displayed no significant difference among the three groups (*P* > 0.05). However, the need for postoperative rescue analgesics was different among the three groups (*P* < 0.05).

**Conclusion:**

The longer the duration of chronic pain was, the higher the incidence of moderate to severe acute postoperative pain was and the greater the amount of analgesics required after surgery.

**Trial registration:**

This study was registered at the Chinese Clinical Trial Registration Center (http://www.chictr.org.cn/index.aspx, clinical trial number: ChiECRCT20200165, date of registration: July 6, 2020).

## Background

Low back pain is one of the most frequent reasons people seek medical services [1]. It not only causes a high disability rate but also increases people’s medical burden [2]. Some people can recover naturally or through some form of intervention, and some people develop chronic low back pain [3]. Chronic low back pain is generally considered to be persistent if it lasts for more than 3 months [4]. The cause of chronic low back pain is uncertain (nonspecific). Some authors have suggested that lumbar spine stenosis, lumbar spondylolisthesis, disc herniation, and degenerative disc disease may play a role in the symptoms [5]. At the same time, these lumbar degenerative diseases are also the most common reasons for patients to choose fusion and internal fixation surgery [6].

In the past two decades, the number of patients choosing lumbar fusion surgery due to lumbar degenerative diseases has been increasing worldwide [7]. Clinical experience highlights that most patients choose fusion surgery to enable them to continue to work and to live an active life [8]. However, spine surgery patients are at high risk of postoperative pain [9]. Optimizing postoperative pain management can facilitate surgical recovery [10]. Risk factors for acute or persistent pain after lumbar fusion surgery can be targeted for perioperative treatment, including tailored postoperative pain treatment or individualized postoperative follow-up. At present, factors affecting the postoperative pain of patients can be broadly grouped into sex [11], obesity [12], preoperative opioid abuse [13], genetics [14], and many other factors. Preoperative chronic pain also affects postoperative pain and postoperative patient functional recovery [15, 16]. However, a careful review of the relevant literature did not yield any report about an association between the duration of chronic low back pain and acute postoperative pain while undergoing lumbar fusion surgery.

Therefore, in the present study, we investigated whether the duration of chronic low back pain influences the acute postoperative pain of patients undergoing lumbar fusion surgery. The visual analogue scale (VAS) is a tool used to assess postoperative pain. Our aim was to provide a reference for the pain management of patients with chronic low back pain.

## Materials and methods

### Ethics and patients

This study was approved by the ethics committee of the Chinese Ethics Committee of Registering Clinical Trials with approval number ChiECRCT20200165. The participating patients provided written informed consent. All methods were carried out in accordance with the Declaration of Helsinki and with the STROBE recommendations. This study was registered at the Chinese Clinical Trial Registration Center under the number ChiCTR2000029923 before patient enrolment. After obtaining written informed consent from all patients, we conducted this prospective observational study at The First Affiliated Hospital of USTC from July 8, 2020, to December 1, 2020. The inclusion criteria were as follows: patients with American Society of Anaesthesiologists (ASA) grades I to III, age range of 18 to 75 years, chronic low back pain supported by a focused history, physical examination and lumbar imaging by X-ray, magnetic resonance imaging, or computed tomography (showing spinal stenosis, spondylolisthesis or disc herniation). These preoperative examinations were conducted by clinicians. Patients with low back pain unresponsive to conservative treatment for more than 3 months and planned to undergo two-level open lumbar fusion surgery under general anaesthesia (all patients underwent the same type of surgery) were included. The exclusion criteria were as follows: patients who refused to participate or who had cognitive impairment (unable to provide informed consent); other chronic pain conditions (not related to surgical indications); took analgesic drugs before surgery (gabapentinoids and/or NSAIDS) or had a history of opioid abuse; chronic low back pain not due to spinal stenosis, spondylolisthesis; or disc herniation but manifested by one or more specific causes (for example, cancer, fractures, and infections); previously underwent lumbar spine surgery; chronic opioid or gabapentin treatment or a visual analogue scale score of > 3 over the past week (The preoperative pain score of the patients was assessed as a resting state. If the preoperative pain score was > 3, the patients were treated with additional analgesic measures, such as analgesic drugs in the hospital, we excluded patients in this category).

### Methods and data acquisition

Patients were evaluated from hospital admission until 3 months after discharge by the same investigator who was blinded to the design of the trial. The evaluator introduced the questionnaire and scoring rules to each patient in detail. The online questionnaire was used to record the clinical information via a tablet. Before surgery, the age, sex, smoking history, body mass index (BMI), operation history, duration of surgery, level of preoperative pain (VAS, the preoperative pain score of the patients was assessed as a resting state), aetiology of back pain (spinal stenosis, spondylolisthesis, or disc herniation), Self-rating Anxiety Scale (SAS), Self-rating Depression Scale (SDS), FRAIL scale, and duration of chronic low back pain were recorded online by an anaesthetis. According to their chronic low back pain duration, the patients could be classified into three groups: duration of pain less than 1 year, one to 5 years, and more than 5 years. We conducted a unified general anaesthesia program. The VAS (ranging from 0 to 10) was used to evaluate the acute postoperative pain (maximum intensity) during the day (VAS day) and night (VAS night) on the first postoperative day after surgery. All patients were treated with postoperative intravenous self-controlled analgesia (PCIA). The PCIA contained 100 μg of sufentanil and 98 mL of saline. The analgesic pump was set as follows: background infusion of sufentanil 2 μg/h, bolus dose of sufentanil 2 μg, and lockout interval 10 min. Patients pressed the PCIA when they experienced pain at rest > 3. If patients still reported pain or greater than 4 on a 0–10 scale on the VAS, supplemental rescue boluses of intravenous flurbiprofen axetil injection of 50 mg were administered. The patient’s analgesic dosage (analgesics for remedy), pain scores on the day of discharge, pain scores after 3 months, and hospital stay were recorded.

### Sample size calculation

To achieve a sufficient sample size, we used PASS software to calculate the power. We first conducted a preliminary experiment, and the means of the maximum pain score (VAS) of the three groups (duration of pain less than 1 year, one to 5 years, and more than 5 years) during the day were 2.60, 2.90, and 3.20, respectively. The SDs of the maximum pain score of the three groups during the day were 0.88, 0.85, and 0.83, respectively. The significance level (α) was set at 0.05 and power (1-β) = 0.98. In the one-way ANOVA in PASS software, the sample size required for each group was 79. The mean maximum pain scores (VAS) of the three groups during the night were 3.55, 3.85, and 4.10, respectively. The SDs of the maximum pain score of the three groups during the day were 0.95, 0.88, and 0.85, respectively. The sample size was calculated in the same way, and the number of patients required for each group was 101. Considering a certain loss to follow-up, the sample size of each group was set to 110.

### Questionnaire and scores

The preoperative mental state was evaluated on the day before surgery using the Chinese-language versions of the SAS and SDS. The SAS and SDS are self-report questionnaires that provide a comprehensive assessment of the anxiety and depression status of patients [17]. The SAS and the SDS comprise 20 items. Each item is scored on a 4-point scale (1– not at all; 2 –somewhat; 3– moderately so; and 4 – very much so); the total score for each questionnaire is multiplied by 1.25 to convert it into a standardized score (range, 25–100; higher scores indicate higher levels of anxiety and depression) [18].

The VAS (Visual Analogue Score) is a tool used to assess pain levels [19]. It is on a scale ranging from 0 (no pain) to 10 (unimaginable pain), mild (0–3), moderate (4–6) and severe pain (7–10), in which higher scores indicate more pain [20].

The FRAIL scale [21, 22] is a simple tool to evaluate the physical condition and includes five items. It measures fatigue, resistance (ability to climb one flight of stairs), ambulation (ability to walk one block), illness (more than five past or current diagnoses), and weight loss (more than 5%). Each positive response within a domain scored 1 point, yielding a maximum score of 5. Higher scores indicate increased frailty [23].

### Statistical analysis

Statistical calculations were performed using IBM SPSS Statistics Version 22. Descriptive analysis was performed to identify the number and percentage of demographic characteristics. Data are shown as the mean ± standard deviation (SD) or number (%). One-way ANOVA and the chi-square test were used to identify the differences among the three groups in the baseline demographic variables (age, sex, BMI, operation history, smoking, level of preoperative pain (VAS), SAS scale, SDS scale, FRAIL scale, duration of surgery, aetiology of back pain). Bivariate correlation analysis [24] (Spearman’s correlation and Pearson’s correlation) was used to estimate the interrelation between some preoperative variables (age, sex, BMI, operation history, smoking, level of preoperative pain (VAS), SAS scale, SDS scale, FRAIL scale, duration of surgery, aetiology of back pain) and the VAS day or VAS night. The independent variables (SAS scale, SDS scale, duration of surgery, aetiology of back pain) with *P* < 0.1 in the bivariate correlation analysis were included in the binary logistic regression for further analysis. Logistic regression models were used to examine the effect of some independent variables on acute postoperative pain (VAS day and VAS night). According to the degree of postoperative pain scores, we converted the measurement data into dichotomous data [25]. Two separated multivariable logistic regression models were built with VAS day and VAS night as outcome respectively. All possible risk factors were tested in univariate logistic regression and then candidates (with *p* < 0.20 in univariate logistic regression) put into the multivariable logistic regression model. After the stepwise model selection, only risk factors with *p* < 0.10 were kept in the final logistic regression model. Odds ratios with 95% confidence intervals were reported based on the regression models. A significant difference between different groups and a significant correlation of variables were set at *P* < 0.05.

## Results

### Patient demographics

Age, sex, BMI, level of preoperative pain (VAS), operation history, smoking history, duration of surgery, SAS, SDS, aetiology of back pain, and FRAIL scale were all recorded as the baseline before surgery. Initially, 332 patients consented to participate in this prospective trial but. 1 patient was lost to follow-up and, 1 patient changed to a different surgical procedure. Data from the remaining 330 patients were included in this analysis. The number and percentage of these items were used to describe and measure the patient demographics in the three groups, as shown in Table 1. Except for the level of preoperative pain, SAS, and SDS, the three groups displayed no significant difference in demographic characteristics or clinical information.

**Table 1:** Summary statistics of the baseline characteristics

Variable	Chronic pain duration classification			*P* value
	< 1 year	1 ~ 5 years	> 5 years	
	(*N* = 110)	(*N* = 110)	(*N* = 110)	
Age, yr (mean ± SD)	54.48 ± 9.94	55.06 ± 10.30	56.36 ± 8.68	0.320
Female (%)	67 (60.91)	64 (58.18)	69 (62.73)	0.482
BMI (mean ± SD)	24.48 ± 3.11	24.12 ± 3.77	24.03 ± 3.67	0.619
Operation history (%)				0.788
0	30 (27.27)	33 (30.00)	31 (28.18)	
1	47 (42.73)	41 (37.27)	46 (41.82)	
≥ 2	33 (30.00)	36 (32.73)	33 (30.00)	
Smoking (%)	40 (36.36)	43 (39.09)	39 (35.45)	0.359
Level of preoperative pain (VAS) (mean ± SD)	1.71 ± 0.65	1.88 ± 0.69	2.07 ± 0.71	< 0.05*
SAS (mean ± SD)	36.91 ± 6.72	40.03 ± 6.59	43.25 ± 6.36	< 0.05*
SDS (mean ± SD)	39.31 ± 7.21	42.35 ± 6.83	46.25 ± 7.28	< 0.05*
FRAIL (mean ± SD)	1.71 ± 0.70	1.66 ± 0.76	1.74 ± 0.75	0.760
Duration of surgery, min (mean ± SD)	121.01 ± 8.69	122.99 ± 9.40	123.75 ± 10.69	0.088
Etiology of back pain(%)				0.817
spinal stenosis	57 (51.82)	51 (46.36)	49 (44.55)	
spondylolisthesis	34 (30.91)	37 (33.67)	41 (37.27)	
disc herniation	19 (17.27)	22 (19.97)	20 (18.18)	

One-way ANOVA and chi-square tests were used to identify the differences among the three groups for some variables

*BMI* Body mass index, *VAS* Visual analogue scale for pain, *SAS* Self-rating Anxiety Scale, *SDS* Self-rating Depression Scale, *FRAIL* FRAIL scale, *SD* Standard deviation, *yr* Year

*P < 0.05; significant difference among the three groups

### Bivariate correlation analysis of factors correlated with acute postoperative pain (VAS scores)

Bivariate correlation analysis was employed to assess the relationship between acute postoperative VAS scores (VAS day and VAS night) and the duration of chronic low back pain or other variables. In the bivariate correlation analysis, age, sex, BMI, level of preoperative pain (VAS), operation history, smoking history, duration of surgery, SAS, SDS, FRAIL scale, aetiology of back pain, and duration of chronic low back pain (three groups) were included. Ultimately, the independent variables with *P* < 0.1 were level of preoperative pain (correlation coefficient: 0.386, *P* < 0.001), SAS (correlation coefficient: 0.735, P < 0.001), SDS (correlation coefficient: 0.734, *P* < 0.001), and duration of chronic low back pain (correlation coefficient: 0.460, *P* < 0.001), which were correlated with VAS day. The independent variables with *P* < 0.1 were level of preoperative pain (correlation coefficient: 0.391, *P* < 0.001), SAS (correlation coefficient: 0.766, P < 0.001), SDS (correlation coefficient: 0.799, P < 0.001), and duration of chronic low back pain (correlation coefficient: 0.397, P < 0.001), which were correlated with VAS night (Table 2).

**Table 2:** Bivariate analysis of factors correlated with VAS day and VAS night

Independent variables			Dependent variables	
	VAS day		VAS night	
	Correlation coefficient	*P* value	Correlation coefficient	*P* value
Age	0.056	0.309	0.041	0.456
Sex	0.048	0.381	−0.037	0.502
BMI	−0.080	0.145	−0.009	0.874
Operation history	−0.004	0.939	−0.056	0.313
Smoking	−0.0077	0.162	0.035	0.525
Level of preoperative pain (VAS)	0.386	< 0.001*	0.391	< 0.001*
SAS	0.735	< 0.001*	0.766	< 0.001*
SDS	0.734	< 0.001*	0.799	< 0.001*
FRAIL	0.012	0.822	0.05	0.365
Duration of surgery	0.064	0.245	0.102	0.064
Chronic pain duration	0.460	< 0.001*	0.397	< 0.001*
Aetiology of back pain	−0.55	0.319	0.023	0.672

Spearman’s correlation analysis and Pearson’s correlation analysis were used in the bivariate analyses. There was a significant difference between the independent variables and the dependent variables

*P < 0.1 (*n* = 330)

*BMI* Body mass index, *VAS* Visual analogue scale for pain, *SAS* Self-rating Anxiety Scale, *SDS* Self-rating Depression Scale, *FRAIL* FRAIL scale

### Logistic regression model to examine the effect of the variables on acute postoperative pain

In the regression model with VAS day had moderate or severe pain score as outcome, only SAS, SDS, duration of chronic low back pain, BMI and aetiology of back pain are included in the multivariable logistic regression. Although the level of preoperative pain (VAS) showed statistical significance in univariate analysis, but it was not included in the final multivariable model, it could be due to the interactions or correlations among variables. SAS and SDS showed statistical significance in both univariate logistic regression and multivariable logistic regression. After controlling SAS, SDS, BMI, and aetiology of back pain, the duration of chronic low back pain did not show statistical significance. The result is uncertain because the confidence interval included the null after controlling for SAS SDS, BMI, and aetiology of back pain (OR = 2.48, 95% CI: 0.900 to 6.828, *p* = 0.0789) (Table 3).

**Table 3:** Univariate and multivariable logistic regression results with VAS day as outcome

Risk factors	Univariate logistic regression		Multivariable logistic regression	
	OR (95% CI)	*p* value	OR (95% CI)	*p* value
SAS	1.581 (1.420, 1.761)	<.0001	1.335 (1.149, 1.551)	0.0002
SDS	1.487 (1.353, 1.634)	<.0001	1.214 (1.070, 1.377)	0.0026
Chronic pain duration				
< 1 year (reference)	1	NA	1	NA
1–5 years	1.863 (0.921, 3.765)	0.0833	1.053 (0.367, 3.020)	0.924
> 5 years	6.333 (3.272, 12.259)	<.0001	2.48 (0.900, 6.828)	0.0789
BMI	0.951 (0.888, 1.019)	0.1548	0.865 (0.766, 0.977)	0.0199
Aetiology of back pain				
Spinal stenosis	2.453 (1.152, 5.222)	0.02	4.252 (1.314, 13.762)	0.0157
Spondylolisthesis	2.364 (1.077, 5.187)	0.0319	5.149 (1.514, 17.512)	0.0087
Disc herniation (reference)	1	NA	1	NA
Preoperative pain score (VAS)	2.574 (1.774, 3.734)	<.0001		
Age	1.019 (0.994, 1.046)	0.1379		
Gender (male)	0.860 (0.526, 1.405)	0.5465		
Duration of surgery	1.019 (0.995, 1.045)	0.1274		
Smoking	1.166 (0.709, 1.918)	0.5449		
Frail	1.063 (0.768, 1.471)	0.7134		
Operation history	1.025 (0.752, 1.398)	0.8762		

Variables for the multivariable logistic regression included SAS, SDS, duration of chronic low back pain, BMI and aetiology of back pain

*BMI* Body mass index, *VAS* Visual analogue scale for pain, *SAS* Self-rating Anxiety Scale, *SDS* Self-rating Depression Scale, *FRAIL* FRAIL scale, *CI* Confidence interval, *OR* Odds ratio, *NA* Not available

The model for VAS night is a little bit different. Only SAS, SDS, and duration of chronic low back pain are included in the multivariable logistic regression. Again, the level of preoperative pain (VAS) showed statistical significance in univariate analysis, but it was not included in the final multivariable model. SAS and SDS still showed statistical significance in both univariate logistic regression and multivariable logistic regression. After controlling SAS and SDS, the duration of chronic low back pain is showed statistically significant. Patients with a duration of chronic low back pain of more than 5 years were more likely having moderate to severe acute postoperative pain (VAS > 3) compared to patients with a duration of chronic low back pain less than 1 year (OR = 3.546, 95% CI: 1.405 to 8.95, *p* = 0.0074) (Table 4).

**Table 4:** Univariate and multivariable logistic regression results with VAS night as outcome

Risk factors	Univariate logistic regression		Multivariable logistic regression	
	OR (95% CI)	*p* value	OR (95% CI)	*p* value
SAS	1.579 (1.429, 1.746)	<.0001	1.273 (1.112, 1.457)	0.0005
SDS	1.488 (1.372, 1.614)	<.0001	1.244 (1.109, 1.396)	0.0002
Chronic pain duration				
< 1 year (reference)	1	NA	1	NA
1–5 years	1.803 (1.055, 3.080)	0.0311	1.069 (0.453, 2.522)	0.8781
> 5 years	7.011 (3.781, 13.002)	<.0001	3.546 (1.405, 8.951)	0.0074
BMI	1.004 (0.943, 1.068)	0.908		
Aetiology of back pain				
Spinal stenosis	0.924 (0.511, 1.671)	0.7925		
Spondylolisthesis	1.389 (0.737, 2.617)	0.3099		
Disc herniation (reference)	1	NA		
Preoperative pain score (VAS)	2.962 (2.064, 4.253)	<.0001		
Age	0.996 (0.973, 1.018)	0.7013		
Gender (male)	1.325 (0.844, 2.081)	0.221		
surgery time	1.018 (0.995, 1.041)	0.1349		
smoking 0	0.707 (0.447, 1.118)	0.1381		
Frail	1.098 (0.814, 1.481)	0.5404		
Operation history	0.898 (0.676, 1.195)	0.4615		

Variables for the multivariable logistic regression included SAS, SDS, and duration of chronic low back pain

*BMI* Body mass index, *VAS* Visual analogue scale for pain, *SAS* Self-rating Anxiety Scale, *SDS* Self-rating Depression Scale, *FRAIL* FRAIL scale, *CI* Confidence interval, *OR* Odds ratio, *NA* Not available

**Fig. 1:**
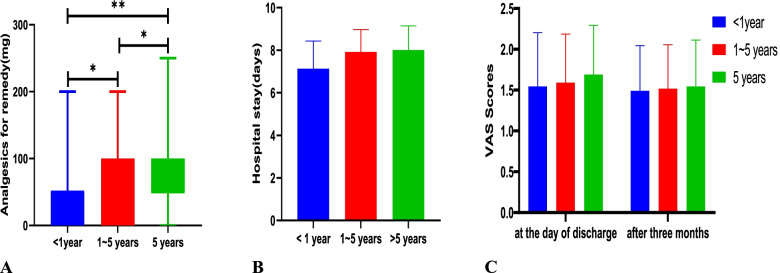
Consumption of postoperative analgesics, hospital stay, pain scores at the day of discharge and after three months among the three groups. Comparisons among groups were performed using analysis of variance and the Kruskal–Wallis test. **indicates *P* < 0.001, * indicates *P* < 0.05

Except for the consumption of analgesics after surgery (Fig. 1A, *P* < 0.05), hospital stay (Fig. 1B), pain scores at the day of discharge and after 3 months displayed no significant difference (Fig. 1C) among the three groups.

## Discussion

The purpose of this study was to examine whether the duration of chronic low back pain before lumbar fusion surgery affected patients’ postoperative pain, consumption of analgesic drugs and hospital stay. The results of our study indicated that the duration of chronic low back pain affected patients’ acute postoperative pain and consumption of analgesic drugs. Postoperative pain is the main factor affecting the functional recovery of patients after surgery [26, 27]. At present, the focus is more on the prevention and treatment of postoperative acute pain [28], often neglecting the impact of preoperative chronic pain on postoperative pain and functional recovery. Chronic pain before surgery causes a series of problems for patients after surgery. Preoperative chronic pain was shown to distract people’s attention before surgery and reduced their recovery of attention and memory abilities during the follow-up period after surgery among nonelderly patients [29].

Doctors have begun to pay more attention to the impact of chronic pain before surgery on postoperative recovery. A prospective study of postoperative pain and functional recovery of patients undergoing hip replacement surgery found that patients with chronic pain other than hip-related pain had slower postoperative mobilization, worse physical function, and greater psychological distress after surgery [15]. Regarding pain sensitivity, researchers have pointed out that patients with chronic low back pain before surgery showed increased sensitivity to pain and decreased sensitivity to harmless stimuli [30], and patients with a history of low back pain had decreased pain tolerance [31]. In a study of the correlation between chronic pain and pain sensitivity, Joachim Erlenwein and his colleagues [32] found that patients with chronic pain had a higher intensity of zoster-related acute pain. However, there is no relevant research on whether the duration of chronic pain affects postoperative pain, consumption of analgesic drugs, or hospital stay. This observational study was the first to report the relationship between the duration of chronic low back pain and acute postoperative pain measured by VAS scores. This study found that the longer the duration of chronic low back pain was, the higher the incidence of moderate to severe acute postoperative pain and the more analgesic drugs were consumed.

Intervertebral disc degeneration is considered to be the main cause of chronic low back pain [33]. Degenerated intervertebral discs contain high levels of proinflammatory mediators and cytokines [34, 35]. Secondary osteoarthritis with or without synovial facets is the main source of chronic low back pain. Long-term inflammation can lead to chronic pain. At the same time, these inflammatory mediators can also reduce the pain threshold of patients [36]. We believe that the impact of chronic pain duration on the acute postoperative pain of lumbar fusion surgery may be related to the duration of chronic inflammation. In a trial [12] that studied obesity and postoperative pain sensitivity, it was found that obese patients had higher pain sensitivity, which may be related to macrophages. Macrophage accumulation is related to the release of inflammatory mediators. Obese patients have more macrophages than nonobese patients, which may help reduce the pain threshold of obese patients [37]. Chronic inflammation is a long-term accumulation process. The longer the duration of chronic pain is, the higher the pain sensitivity after lumbar fusion surgery, which may be related to the release of more inflammatory mediators in these patients.

Another reason may be that the duration of chronic pain is related to different mental and psychological effects on patients. Chronic pain is often accompanied by psychosocial complications [38]. Preoperative anxiety levels were associated with the efficacy of pain management after knee surgery [14], and the more severe the preoperative anxiety was, the greater the need for analgesia was [39]. Our research also shows that the longer the duration of CLBP, the higher the degree of anxiety and depression in patients. Chronic pain can lead to the development and aggravation of anxiety disorders, whereas anxiety disorders can lead to increased pain duration and intensity [40]. This shows that chronic pain has a series of effects on the patient’s emotions and psychology. The longer the chronic pain lasts, the more likely it is that the patient will have symptoms, such as anxiety.

Research [41] has found changes in anxiety and depression was associated with pain severity undergoing lower-extremity total joint arthroplasty. This was consistent with our conclusion. However, for some previously reported factors associated with pain sensitivity such as female sex [42], history of tobacco use [43], our results did not suggest an association.

However, this study also has some limitations. First, as lumbar disc herniation is a naturally occurring disease related to the patient’s lifestyle, the duration of chronic low back pain, which is provided by patients, may not be accurate and thus this might undermine the results. Therefore, we also specifically divided the chronic pain time into three groups to reduce the impact of this potentially inaccurate information on the results. Second, there were other important factors (e.g., disability, education, living status, insurance claims) not reported in our study, and these factors may be associated with acute postoperative pain intensity. Third, this is a single-centre, relatively small population trial, and a multicentre trial is necessary to verify the results.

## Conclusion

For patients with a history of chronic low back pain who underwent lumbar fusion surgery, the longer the preoperative pain lasted, the higher the incidence of moderate to severe acute postoperative pain and the more analgesic drugs required after surgery. Therefore, it is very important to pay attention to patients’ chronic pain before surgery, especially the duration of chronic pain. Patients with long-lasting chronic pain should undergo stricter analgesia treatment supervision after surgery to prevent them from suffering from more severe pain.

## Data Availability

The datasets used and/or analysed during the current study are available from the corresponding author upon reasonable request. The datasets will be available at www.chictr.org.cn 6 months after publication.

## Abbreviations

BMI: Body mass index
VAS: Visual analogue scale for pain
SAS: Self-rating Anxiety Scale
SDS: Self-rating Depression Scale
FRAIL: FRAIL scale
SD: Standard deviation
